# Effect of Ammonia on the Gas-Phase Hydration of the Common Atmospheric Ion HSO_4_^−^

**DOI:** 10.3390/ijms9112184

**Published:** 2008-11-07

**Authors:** Alexey B. Nadykto, Fangqun Yu, Jason Herb

**Affiliations:** Atmospheric Sciences Research Center, State University of New York at Albany, 251 Fuller Rd., Albany 12203, NY, USA

**Keywords:** Nucleation precursors, sulfuric acid, ammonia, hydration thermochemistry, ion-mediated nucleation

## Abstract

Hydration directly affects the mobility, thermodynamic properties, lifetime and nucleation rates of atmospheric ions. In the present study, the role of ammonia on the formation of hydrogen bonded complexes of the common atmospheric hydrogensulfate (HSO_4_^−^) ion with water has been investigated using the Density Functional Theory (DFT). Our findings rule out the stabilizing effect of ammonia on the formation of negatively charged cluster hydrates and show clearly that the conventional (classical) treatment of ionic clusters as presumably more stable compared to neutrals may not be applicable to pre-nucleation clusters. These considerations lead us to conclude that not only quantitative but also qualitative assessment of the relative thermodynamic stability of atmospheric clusters requires a quantum-chemical treatment.

## 1. Introduction

The nucleation of condensable vapours in the Earth atmosphere is critically important for the atmospheric aerosol formation associated with the aerosol radiate forcing and global climate changes [1–3]. The dominant constituent of condensable vapours in the atmosphere is water, which is incapable of self-nucleation due to very low supersaturations under typical atmospheric conditions. The atmospheric nucleation is multicomponent process, in which sulfuric acid plays a role the key atmospheric nucleation precursor. The presence of sulfuric acid allows the formation of binary H_2_SO_4_^−^H_2_O clusters, which are more stable compared to unary water clusters, and can grow into critical embryos under favorable ambient conditions. The critical role of sulfuric acid in atmospheric nucleation is commonly accepted; however, the binary homogeneous nucleation of sulfuric acid and water (BHN) [4–5] is incapable of explaining observed nucleation events in the lower troposphere. Other proposed nucleation mechanisms (a) ternary homogeneous nucleation (THN) of H_2_SO_4_^−^H_2_O- NH_3_ [6–7]; (b) ion-mediated nucleation (IMN) of H_2_SO_4_^−^H_2_O-Ion [8]; and (c) organics-enhanced nucleation H_2_SO_4_^−^H_2_O-organics [9,10]; also involve sulfuric acid and water. The role of ammonia, which was suggested as a principle stabilizer of H_2_SO_4_^−^H_2_O clusters in the atmosphere in the 1990s, remains controversial. Although ammonia is capable of neutralizing aqueous solutions of sulfuric acid, its efficiency in stabilizing binary sulfuric acid-water clusters remains unclear. THN [6], which is based on the classical liquid droplet formalism, predict NH_3_ at ppt level to enhance nucleation rates by ∼30 orders of magnitude. However, predictions of classical THN contradict to both the existing laboratory studies [11–14], and the kinetically-consistent THN model constrained by experimental data [7]. Both experimental data and kinetically consistent THN indicate that the presence of NH_3_ at ppb - ppm levels enhances the H_2_SO_4_^−^H_2_O nucleation by up 10^2^ only. Quantum-chemical studies have indicated that the presence of ammonia leads to a modest enhancement in the stability of H_2_SO_4_^−^ H_2_O clusters; however, they are likely to rule out the exclusive role of ammonia in the atmosphere because more abundant low molecular organic acids (formic acid, acetic acid) were found to enhance the stability of H_2_SO_4_^−^ H_2_O nearly as well as NH_3_ [10].

Atmospheric ions appear to be involved in most of the nucleation events observed in boreal forests [15–18]; however, the relative importance of IMN and other nucleation mechanisms is still a subject of on-going debates [18]. The hydration is a fundamental phenomena that directly affects the ion mobility, stability, lifetime and nucleation rates. The reduction of uncertainties in nucleation calculations requires a clear understanding of the hydration thermodynamics and role of ammonia in the hydrate formation. While structure and properties of neutral (H_2_SO_4_) (NH_3_) (H_2_O)_n_ clusters have been studied [e.g. 10, 20, 21] ; the information concerning the role of ammonia in the formation of ionic clusters containing sulfuric acid, ammonia and water is limited. No data for positives are available at the present time. and the only available data for negatives [22, 23] are limited to (HSO_4_^−^) (NH_3_) and (HSO_4_^−^) (NH_3_) (H_2_O).

In the present Communication, the effect of ammonia on the thermochemical stability of common atmospheric hydrogensulphate (HSO_4_)^−^ ion has been investigated. The structure, properties and thermochemical stability of the gas-phase hydrate clusters (HSO_4_^−^)(NH_3_)(H_2_O)_n_ (n = 1–5) have been studied using the Density Functional Theory. The thermochemical analysis of the relative cluster stability has been carried out, and the involvement of ammonia in the formation of negatively charged sulfuric acid-water clusters under the atmospheric conditions has been discussed. The new thermochemical data that can be utilized directly for the kinetic IMN calculations have been reported, and the atmospheric implications of the obtained results have been discussed.

## 2. Results and Discussion

### 2.1. Structure and geometric properties

Initial generated structures were treated initially by a semi-empirical PM3 [24] method and then by PW91PW91/6–31+G*. Finally, the most stable (within ∼4 kcal/mol from the lowest energy isomer) structures obtained at PW91PW91/6–31+G* level have been optimized at PW91PW91/6–311++G(3df.3pd) level. PW91PW91/6–311++G(3df,3pd) has been used to obtain both equilibrium geometries and thermochemical properties from computed vibrational spectrums. The previous applications of the PW91PW91 method [25] for studying clusters composed of atmospheric species have shown that the aforementioned density functional is capable of providing good geometries, excellent vibrational frequencies in harmonic approximation and quite accurate free energies [10, 19–21, 27–31]. An interested reader can find the discussion about anharmonic effects for atmospheric clusters in the recent review [21]. The PW91PW91 density functional has been used in combination with the largest Pople basis set 6–311++G(3df,3pd) [26] that provides quite small basis set superposition error (BSSE) (e.g. [22, 32]).

**Figure 1.**
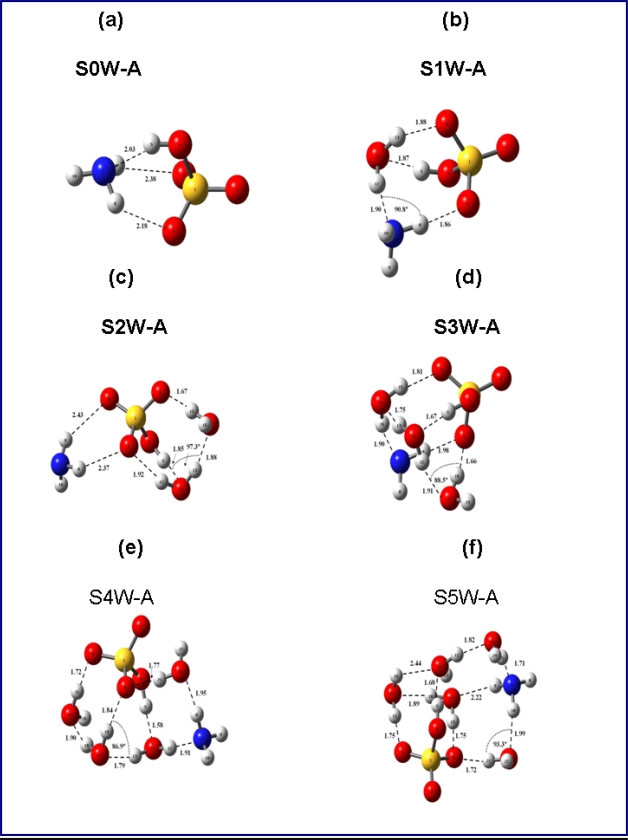
Most stable isomers of (a) (HSO_4_^−^)(NH_3_); (b) (HSO_4_^−^)(NH_3_) (H_2_O); (c) (HSO_4_^−^)(NH_3_)(H_2_O)_2_; (d) (HSO_4_^−^)(NH_3_)(H_2_O)_3_; (e) (HSO_4_^−^)(NH_3_)(H_2_O)_4_; (f) (HSO_4_^−^)(NH_3_)(H_2_O)_5_ obtained at PW91PW91/6–311++G(3df,3pd) level of theory.

Figure 1 presents the equilibrium geometries of the most stable isomers of (HSO_4_^−^)(NH_3_)(H_2_O)_n_.

As seen from Figure 1(a) and Table 1, the structures of (HSO_4_^−^)(NH_3_) obtained at PW91PW91/6–311++G(3df,3pd) (PW91) and MP2/aug-cc-pv(D+d)z with MP2/aug-cc-pV(T+d)Z and MP4/aug-cc-pV(D+d)Z energy corrections to the MP2/aug-cc-pV(D+d)Z geometry levels of theory are similar. Bonding lengths and angles agree within ∼ 2–4% and 0.7–5%, respectively.

### 3.2. Thermochemical Properties

**Figure 2.**
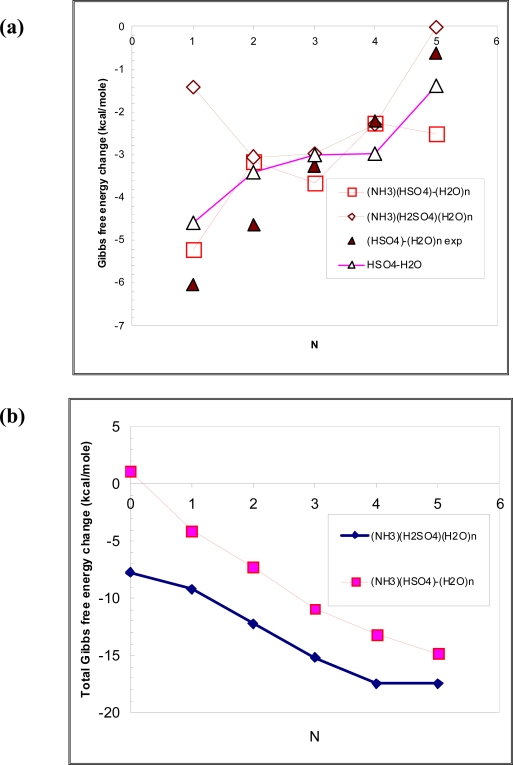
The comparison of: (a) the stepwise Gibbs free energy changes associated with the hydration of (HSO_4_^−^)(H_2_O)_n._ [35], (H_2_SO_4_)(NH_3_)(H_2_O)_n_ [21] and (HSO_4_^−^)(NH_3_)(H_2_O)_n_ (present study) and (b) total free energies associated with the formation of (H_2_SO_4_)(NH_3_)(H_2_O)_n_ [21] and (HSO_4_^−^)(NH_3_)(H_2_O)_n_ from (H_2_SO_4_), (NH_3_) and water molecules and (H_2_O)_n_ and (HSO_4_^−^), (NH_3_) and water molecules, respectively. T = 298.15 K and P = 101.3 KPa. Subscript “exp.” refers to [34].

The growing interest to the thermochemistry of atmospheric clusters is related to the very high sensitivity of nucleation rates to the thermochemistry of first few steps of the cluster formation. All the data are given at standard conditions. The value for other conditions can be obtained using the mass action law. Tables 2 and 3 present calculated hydration enthalpies, entropies and Gibbs free energies associated with the formation of (HSO_4_^−^)(NH_3_)(H_2_O)_n_ by addition of water and ammonia, respectively. Figure 2 presents the comparison of formation and stepwise hydration free energies for (HSO_4_^−^)(H_2_O)_n,_ (HSO_4_^−^)(NH_3_)(H_2_O)_n_ and (H_2_SO_4_)(NH_3_)(H_2_O)_n_.

As seen from Table 2 and Figure 2(a), the presence of ammonia does not lead to a noticeable enhancement in the hydration strength. As may be seen from Figure 2(a), the hydration free energies of (HSO_4_^−^)(H_2_O)_n_ and (HSO_4_^−^)(NH_3_)(H_2_O)_n_ are close, although the hydration of (HSO_4_^−^) (H_2_O)_n_ is somewhat systematically stronger (∼ 0.5 kcal.mol) than that of (HSO_4_^−^)(NH_3_)(H_2_O)_n._ The Gibbs free energies of the initial (n = 1, 2) steps of hydration of (HSO_4_^−^)(NH_3_) is more negative than those of (H_2_SO_4_)(NH_3_); however, the hydration free energies of latter steps are nearly identical. PW91PW91/6–311++G(3df,3pd) and MP2/aug-cc-pv(D+d)z with MP2/aug-cc-pV(T+d)Z and MP4/aug-cc-pV(D+d)Z energy corrections to the MP2/aug-cc-pV(D+d)Z geometry hydration free energies for (HSO_4_^−^)(NH_3_) + H_2_O ↔ (HSO_4_^−^)(NH_3_)(H_2_O) reaction agree within ∼1 kcal mol^−1^.

As seen from Table 3, the affinity of ammonia to (HSO_4_) ^−^ is extremely low (0.7–2.5 kcal mol^−1^) that is 9 kcal mol^−1^ smaller than the affinity of ammonia to neutral H_2_SO_4_. This somewhat surprising finding correlates well with the difference in the structure of (HSO_4_^−^)(NH_3_) and (H_2_SO_4_)(NH_3_), particularly in the intermolecular bonding distances, which are shorter in (H_2_SO_4_)(NH_3_) [21]. The free energies of (HSO_4_ ^−^) + (NH_3_) ⇔ (HSO_4_^−^)(NH_3_) reaction obtained at PW91PW91/6–311++G(3df,3pd) and MP2/aug-cc-pv(D+d)z with MP2/aug-cc-pV(T+d)Z and MP4/aug-cc-pV(D+d)Z energy corrections to the MP2/aug-cc-pV(D+d)Z geometry [22] levels of theory agree within 0.4 kcal mol^−1^.

As may be seen from Figure 2(b), the total change in the Gibbs free energy associated with the formation of (H_2_SO_4_) (NH_3_) (H_2_O)_n_ is larger than that of (HSO_4_^−^)(NH_3_) (H_2_O)_n_ and, thus, the formation of (H_2_SO_4_) (NH_3_) (H_2_O) in the atmosphere is more favorable thermodynamically than the formation of (HSO_4_^−^) (NH_3_) (H_2_O)_n_. Although the hydration of (HSO_4_^−^)(NH_3_) and (HSO_4_^−^)(NH_3_)(H_2_O) is stronger than that of (H_2_SO_4_) (NH_3_) and (H_2_SO_4_) (NH_3_) (H_2_O), the difference of ∼2–3 kcal mol-1 per step is not high enough to compensate a very large ( > 9 kcal mol^−1^) difference in free energy changes between (HSO_4_) ^−^ + (NH_3_) ⇔ (HSO_4_) ^−^ (NH_3_) and (H_2_SO_4_) + (NH_3_) ⇔ (H_2_SO_4_)(NH_3_) reactions.

As seen from Table 4, the presence of additional sulfuric acid does not enhance the affinity of ammonia to negatively charged binary clusters, which remains very low. The presence of ammonia does not lead to any substantial enhancement in the hydration of binary cluster ions or affinity of sulfuric acid to negatively charged binary clusters. These considerations rule out the stabilizing role of ammonia in the formation of negatively charged clusters (HSO_4_^−^)(H_2_O)_n_ and indicate that the assessment of charged clusters as presumably more stable compared to neutrals may be inapplicable to atmospheric pre-nucleation clusters.

## 4. Conclusions

In this paper, the role of ammonia, a commonly accepted principle stabilizer of binary sulfuric acid-water clusters in the atmosphere, in the formation of hydrogen bonded complexes of common atmospheric hydrogensulfate ion (HSO_4_^−^) with water has been investigated. New thermochemical data for the hydration entropies, enthalpies and Gibbs free energies have been reported and the thermodynamic analysis of the hydrate stability has been performed. The results of the present study lead us to the following conclusions:

- The presence of NH_3_ does not enhance the thermochemical stability of HSO_4_^−^ (H_2_O)_n_ and ammonia is unlikely involved in the gas-phase hydration of hydrogensulfate ion under the atmospheric conditions.
- The total free energy change associated with the formation of charged (HSO_4_^−^) (NH_3_) (H_2_O)_n_ is less negative than that associated with the formation of neutral (H_2_SO_4_) (NH_3_) (H_2_O)_n_ due to the very low affinity of NH_3_ towards (HSO_4_^−^). This leads us to conclude that the assessment of charged clusters in the classical nucleation theory as presumably more stable thermodynamically compared to neutrals is not applicable to pre-nucleation ternary clusters, or generally multicomponent molecular clusters. This is a clear indication that not only quantitative, but also qualitative assessment of the relative thermodynamical stability of atmospheric clusters is impossible without the quantum-chemical treatment.

The obtained results can be applied to a wide range of problems related to chemical physics of the atmospheric aerosol formation, chemical technology and air quality research and they can be utilized directly in computations of the hydrate distributions in the atmospheric conditions and kinetic simulations of nucleation rates.

## Figures and Tables

**Table 1.** Geometrical properties (intermolecular distances R(a,b) and angles A(a,b,c)) of (HSO_4_^−^)(NH_3_) obtained at PW91PW91/6–311++G(3df,3pd) (PW91) and MP2/aug-cc-pv(D+d)z [22] levels of theory.

	R(1,2)	R(1,3)	R(1,4)	R(1,6)	R(3,8)	R(4,5)	R(5,7)	R(6,9)	R(7,8)	R(7,9)	R(7,10)
**PW91**	1.46	1.48	1.67	1.48	2.18	0.99	2.03	2.39	1.03	1.02	1.02
**MP2**					2.28		2.08	2.28			
	A	A	A	A	A	A	A	A	A	A	A
	(2,1,3)	(2,1,4)	(3,1,4)	(3,1,6)	(1,3,8)	(4,5,7)	(5,7,9)	(8,7,9)	(9,7,10)	(3,8,7)	(6,9,7)
**PW91**	115.17	102.57	104.56	112.56	97.81	149.58	88.95	103.15	108.55	135.63	123.15
**MP2**						148.90				129.30	129.20

**Table 2.** Enthalpies (kcal mol^−1^), entropies (cal mol^−1^ K^−1^), and Gibbs free energy changes (kcal mol^−1^) of (HSO_4_^−^)(NH_3_)(H_2_O)_n_ hydration calculated at T = 298.15K and P = 101.3 KPa. Superscripts *a* refers to MP2/aug-cc-pv(D+d)Z study [22].

	ΔH	ΔS	ΔG
(H SO_4_^−^ ) (NH_3_)+H_2_O ⇔ (HSO_4_^−^)( NH_3_) (H_2_O)	−15.79	−35.53	−5.20
	−13.07^a^	−30.00^a^	−4.23^a^
(H SO_4_^−^)(NH_3_) (H_2_O) +H_2_O ⇔ (H SO_4_^−^)(NH_3_) (H_2_O)_2_	−12.39	−30.97	−3.16
(H SO_4_^−^) (NH_3_) (H_2_O)_2_ +H_2_O ⇔ (H SO_4_^−^)(NH_3_) (H_2_O)_3_	−14.42	−36.07	−3.67
(H SO_4_^−^)(NH_3_) (H_2_O)_3_ +H_2_O ⇔ (HSO_4_^−^)(NH_3_) (H_2_O)_4_	−10.91	−29.01	−2.25
(H SO_4_^−^)(NH_3_) (H_2_O)_4_ +H_2_O ⇔ (HSO_4_^−^)(NH_3_) (H_2_O)_5_	−12.70	−34.17	−2.51

**Table 3.** Enthalpies (kcal mol^−1^), entropies (cal mol^−1^ K^−1^), and Gibbs free energy changes (kcal mol^−1^) of (HSO_4_^−^)(NH_3_)(H_2_O)_n_ formation by addition of ammonia. T=298.15K and P=101.3 KPa. Superscript *a* refers to MP2/aug-cc-pv(D+d)Z study [22].

	ΔH	ΔS	ΔG
(H SO_4_^−^) +(NH_3_) ⇔ (HSO_4_^−^) (NH_3_)	−7.22	−27.90	1.10
	−9.24^a^	−32.37^a^	0.69^a^
(H SO_4_^−^) (H_2_O)+(NH_3_) ⇔ (H SO_4_^−^) (NH_3_) (H_2_O)	−9.44	−32.41	1.12
(H SO_4_^−^) (H_2_O)_2_+(NH_3_) ⇔ (H SO_4_^−^) (NH_3_) (H_2_O)_2_	−7.24	−28.94	1.39
(H SO_4_^−^) (H_2_O)_3_+(NH_3_) ⇔ (H SO_4_^−^) (NH_3_) (H_2_O)_3_	−9.46	−33.86	0.64
(H SO_4_^−^) (H_2_O)_4_+(NH_3_) ⇔ (H SO_4_^−^) (NH_3_) (H_2_O)_4_	−9.44	−36.47	1.43
(H SO_4_^−^) (H_2_O)_5_+(NH_3_) ⇔ (H SO_4_^−^) (NH_3_) (H_2_O)_5_	−9.07	−31.42	0.30

**Table 4.** Enthalpies (kcal mol^−1^), entropies (cal mol^−1^ K^−1^), and Gibbs free energy changes (kcal mol^−1^) of (HSO_4_^−^) (H_2_SO_4_) (NH_3_)(H_2_O)_n_ and (HSO_4_^−^)(H_2_SO_4_)(H_2_O)_n_ formation. T=298.15K and P=101.3 KPa. Superscript “*a”* refers to [36].

Reaction	ΔH	ΔS	ΔG
(HSO_4_^−^)(H_2_SO_4_) (NH_3_) +H_2_O ⇔ (HSO_4_^−^)(H_2_SO_4_) (NH_3_) (H_2_O)_1_	−8.83	−23.9	−1.7
(HSO_4_^−^)(H_2_SO_4_) +H_2_O ⇔ (HSO_4_^−^)(H_2_SO_4_)(H_2_O)_1_	−8.2^a^		−0.6^a^
(HSO_4_^−^) (NH_3_) +(H_2_SO_4_) ⇔ (HSO_4_^−^)(H_2_SO_4_) (NH_3_)	−46.58	−42.65	−33.86
(HSO_4_^−^) +(H_2_SO_4_) ⇔ (HSO_4_^−^)(H_2_SO_4_)	−45.70^a^		−32.70^a^
(HSO_4_^−^) (NH_3_) (H_2_O)_1_ +(H_2_SO_4_) ⇔ (HSO_4_^−^)(H_2_SO_4_) (NH_3_) (H_2_O)_1_	−39.61	−31.02	−30.37
(HSO_4_^−^) (H_2_O)_1_ +(H_2_SO_4_) ⇔ (HSO_4_^−^)(H_2_SO_4_) (H_2_O)_1_	−40.30^a^		−28.1^a^
(HSO_4_^−^)(H_2_SO_4_) (H_2_O)_0_ +NH_3_ ⇔ (HSO_4_^−^)(H_2_SO_4_) (H_2_O)_0_(NH_3_)	−8.08	−27.01	−0.02
(HSO_4_^−^)(H_2_SO_4_) (H_2_O)_1_ +NH_3_ ⇔ (HSO_4_^−^)(H_2_SO_4_) (H_2_O)_1_(NH_3_)	−8.75	−25.59	−1.12
